# Lessons from a natural experiment: Allopatric morphological divergence and sympatric diversification in the Midas cichlid species complex are largely influenced by ecology in a deterministic way

**DOI:** 10.1002/evl3.64

**Published:** 2018-06-27

**Authors:** Andreas F. Kautt, Gonzalo Machado‐Schiaffino, Axel Meyer

**Affiliations:** ^1^ Department of Biology University of Konstanz Universitätsstraße 10 78457 Konstanz Germany; ^2^ Current Address: Genetics Area, Department of Functional Biology University of Oviedo 33006 Oviedo Spain; ^3^ Radcliffe Institute for Advanced Study Harvard University Cambridge Massachusetts 02138

**Keywords:** Admixture, colonization history, demographic inference, ecological opportunity, evolutionary rate, founder effect, geometric morphometrics, parallel evolution, phenotypic trajectory, RADseq

## Abstract

Explaining why some lineages diversify while others do not and how are key objectives in evolutionary biology. Young radiations of closely related species derived from the same source population provide an excellent opportunity to disentangle the relative contributions of possible drivers of diversification. In these settings, lineage‐specific effects are shared and can be ruled out. Moreover, the relevant demographic and ecological parameters can be estimated accurately. Midas cichlid fish in Nicaragua have repeatedly colonized several crater lakes, diverged from the same source populations, and, interestingly, diversified in some of them but not others. Here, using the most comprehensive molecular and geometric morphometric data set on Midas cichlids to date (∼20,000 SNPs, 12 landmarks, ∼700 individuals), we aim to understand why and how crater lake populations diverge and why some of them are more prone to diversify in sympatry than others. Taking ancestor‐descendant relationships into account, we find that Midas cichlids diverged in parallel from their source population mostly—but not exclusively—by evolving more slender body shapes in all six investigated crater lakes. Admixture among crater lakes has possibly facilitated this process in one case, but overall, admixture and secondary waves of colonization cannot predict morphological divergence and intralacustrine diversification. Instead, morphological divergence is larger the more dissimilar a crater lake is compared to the source lake and happens rapidly after colonization followed by a slow‐down with time. Our data also provide some evidence that founder effects may positively contribute to divergence. The depth of a crater lake is positively associated with variation in body shapes (and number of species), presumably by providing more ecological opportunities. In conclusion, we find that parallel morphological divergence in allopatry and the propensity for diversification in sympatry across the entire Midas cichlid fish radiation is partly predictable and mostly driven by ecology.

**Impact summary**

The variation in diversification rates and associated species richness among organisms is stunning and apparent at different taxonomic levels. For example, almost half of the more than 60,000 described extant species of vertebrates are teleost fishes and of those about 2500, in turn, belong to the family of cichlid fishes (*Cichlidae*). In other words, approximately every 25th vertebrate species is a cichlid fish. Moreover, at an even lower taxonomic scale, it is the Haplochromines that account for the majority of cichlid species diversity in Africa, whereas other cichlid “tribes” are arguably species‐poor in comparison. In this study, we aim to identify factors that explain when and how diversification takes place. We make use of a study system at the lowest level of taxonomic scale. Midas cichlids in Nicaraguan crater lakes form a species complex of more than 11 closely related lineages that have evolved in less than 2000 generations. Stemming from the same source populations, unlike other cichlid lineages in these lakes, all Midas cichlids have morphologically diverged from their source population and even diversified into multiple species within at least two crater lakes without geographic barriers. We find that the ecological environment of a crater lake predicts how different crater lake populations will be from their source population. Furthermore, diversification within crater lakes can probably only occur if enough “ecological opportunity” in the form of water depth and habitat diversity exists. Thus, overall, our study shows that ecology plays a major role in shaping the diversity of an entire species complex—both among and within lakes—in a largely predictable manner.

Understanding why some lineages tend to diversify prolifically while others do not is a key objective in evolutionary biology (Seehausen and Wagner 2014; Grant and Grant 2017). Among the most important factors thought to positively influence diversification rates is ecological opportunity (Schluter 2000; Losos and Ricklefs 2009; Yoder et al. 2010), which can come about either by the evolution of key innovations, the colonization of habitats with underutilized niches, the extinction of previously dominating lineages, or the appearance of new resources (Stroud and Losos 2016). While key innovations (e.g., modification of the pharyngeal jaws in cichlid fishes (Liem 1973)) can be potentially invoked to explain the diversity of a clade in general, they cannot by themselves explain pronounced differences in diversification rates between closely related lineages that also share that particular key innovation. Similarly, the colonization of a new habitat, even if it happened repeatedly within a single lineage, does not always result in divergence and incipient adaptive radiation (Martin 2016). In other words, factors other than ecological opportunity are likely to often influence the onset of any particular adaptive radiation (Stroud and Losos 2016). Historical contingencies, for one, presumably play a large role in most radiations (Gavrilets and Losos 2009). For example, the relative timing of colonization events can determine the fate of radiations (i.e., priority effects): a resident species can potentially prevent the colonization of an ecologically equivalent species or suppress its subsequent diversification (Chase 2007; Tan et al. 2017). Moreover, various demographic factors even within a single colonizing lineage can have diverse effects. In the context of the colonization of a new habitat, the size and growth rate of the founder population will determine the strength of the founder effect, which can, in turn, affect morphological divergence (Mayr 1954; Kolbe et al. 2012). In addition, bouts of secondary colonization and hybridization can facilitate diversification processes by seeding a population with genetic variation (e.g., increasing variation in adaptive traits or mate preferences and incompatibilities) that evolved in a phase of allopatric isolation (Seehausen 2004; Martin et al. 2015; Meier et al. 2017; Richards and Martin 2017).

The increasing complexity of historical processes over large time scales can render disentangling the contributions of various extrinsic and intrinsic factors difficult. In young (or ongoing) adaptive radiations, on the other hand, the ecological conditions driving diversification have presumably often not changed and the demographic histories of populations can still be reconstructed in detail (e.g., Kavembe et al. 2016). Thus, studies of young radiations allow for insights into the immediate factors that drive diversification and can complement large‐scale macroevolutionary studies in important ways (de la Harpe et al. 2017; Recknagel et al. 2017). In particular, study systems with natural replicates (e.g., repeated colonization of islands or lakes) may allow to reveal factors that are positively or negatively associated with diversification rates or a proxy thereof (Losos and Ricklefs 2009; Seehausen and Wagner 2014; Martin 2016). In this regard, diversification rates themselves are often hard to quantify in young systems in which population divergence may be at the earliest stage of the speciation process. A simple determination of the number of species in still radiating systems will likely be subject to taxonomical ambiguities. Morphological variation, on the other hand, has proven to be a useful, quantifiable measure of adaptive diversity (Roy and Foote 1997; Mahler et al. 2010) with species in most adaptive radiations showing differences in morphology (Schluter 2000). In fish, body shape constitutes an adaptive trait. Lacustrine fish often exhibit elongated body shapes in the open‐water zone (limnetic environment) and are usually more deep‐bodied in the benthic environment (Webb 1984; Meyer 1990; Schluter 1993; Langerhans and Reznick 2010; Kautt et al. 2016b).

Midas cichlid fish (*Amphilophus spp*. species complex) in Nicaragua have repeatedly colonized small and remote crater lakes and subsequently diverged from the same source populations and diversified phenotypically in situ in some of them but less so in others (Elmer et al. 2010a; Kautt et al. 2016a). Accordingly, divergence and diversification in Midas cichlids has happened in complete geographic isolation as well as in full sympatry. The two great lakes, Nicaragua and Managua, are intermittently connected by Rio Tipitapa, which flows from L. Managua into L. Nicaragua through Tisma Pond (Fig. 1). Both great lakes harbor the same set of Midas cichlid species, *A. citrinellus* and *A. labiatus*. The former is considered the archetype of the species complex, whereas the latter is characterized by its markedly pronounced hypertrophied lips and a more pointed and narrow head shape (Barlow and Munsey 1976). The two species in the great lakes are not equally abundant, with *A. labiatus* being much rarer; its relative frequency compared to *A. citrinellus* fluctuates from as little as one percent to only around 15 percent.

**Figure 1:**
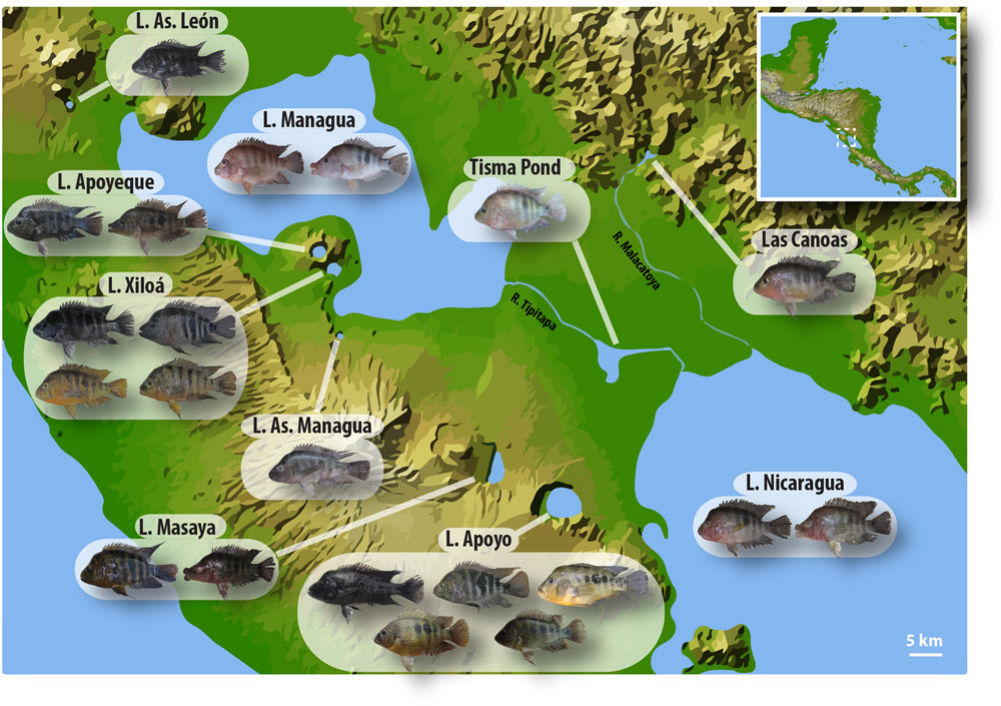
Geographic distribution and morphological diversity of focal populations of Midas cichlid fish. The two great lakes Nicaragua and Managua are intermittently connected by Rio Tipitapa that flows through Tisma Pond. Las Canoas is not a crater lake, but a water reservoir, which had historically been connected to L. Nicaragua by Rio Malacatoya until the construction of a dam. All crater lakes are isolated bodies of water with no inlet or outlet. Superimposed on the map are representative specimens of species and ecotypes inhabiting the lakes.

At least seven crater lakes are known to harbor Midas cichlids, but so far most studies (including this one) have focused on the largest six crater lakes (Barluenga and Meyer 2010; Elmer et al. 2010a; Geiger et al. 2010a), since the remaining tiny Crater Lake Tiscapa is located within the city of Managua and has been highly polluted by anthropogenic activity. Of the six main crater lakes, all have been colonized from L. Managua, except for L. Apoyo that has been colonized from L. Nicaragua (Barluenga and Meyer 2010; Machado‐Schiaffino et al. 2017). No water connections are known to exist between crater lakes. Crater lakes are remarkably deep (mean depth between 17.2 and 142 meters) and are characterized by a large open‐water zone (Waid et al. 1999). They provide thus a generally very different environment from the extremely shallow, but old, source lakes Managua and Nicaragua (mean depth of 8.6 and 12.4 meters) (Elmer et al. 2010a). Apart from the great lakes and crater lakes, Midas cichlids occur in rivers across Nicaragua and a population of Midas cichlids exists in the water reservoir Las Canoas, which was historically connected to L. Nicaragua via Rio Malacatoya until the construction of a dam.

All crater lake populations are morphologically distinct from the source populations (Elmer et al. 2010a), but in only two of the crater lakes, Apoyo and Xiloá, have multiple endemic species of Midas cichlids been described (so far). In L. Apoyo, the six species—described based on morphology—(Barlow and Munsey 1976; Stauffer et al. 2008; Geiger et al. 2010b) are, however, not in concordance with population genetic data that provide evidence for only five genetic clusters (Kautt et al. 2016a). In L. Xiloá, the four described species (Stauffer and McKaye 2002; Recknagel et al. 2013b) are in concordance with four distinct genetic clusters, although hybridization between two of them seems to be still ongoing to a considerable extent (Kautt et al. 2016a). Notably, in both of these lakes a species with an elongated body shape adapted to the open water niche (referred to as “limnetic”) has evolved in parallel (Elmer et al. 2014). Crater lakes Apoyeque and Masaya harbor each a morphologically polymorphic population in which a certain proportion of fish (5–20%) exhibit hypertrophied lips (Elmer et al. 2010b; Machado‐Schiaffino et al. 2017), phenotypically resembling *A. labiatus* from the great lakes. Two of the smallest crater lakes, Asososca Managua and Asososca León, are inhabited by populations with a rather continuous distribution of phenotypes, albeit in the former lake fish may be at the earliest stages of divergence along the benthic‐limnetic axis (Kautt et al. 2016b), similarly to fish in crater lakes Apoyo and Xiloá.

Accordingly, crater lake Midas cichlids represent an interesting natural experiment with populations at different stages of divergence and intralacustrine diversification. This allows to ask: what is driving morphological divergence and speciation after colonization, if it is happening in a deterministic fashion, and why did intralacustrine phenotypic diversification take place only in some crater lakes and not in others. Yet, this can only be tested critically with a large samples size of all populations (Geiger et al. 2010a) and a good understanding of their evolutionary relationships and demographic histories (Simoes et al. 2016). In a previous study, we found a positive correlation between the mean depth of a crater lake and variation in body elongation, and that lakes with a larger littoral zone harbor on average more deep‐bodied individuals (Recknagel et al. 2014). However, without reliable estimates of the colonization history, the effects of demographic correlates on morphological evolution, and its speed, could not be tested. Furthermore, univariate measures of morphology cannot capture differences in the direction of divergence. Multivariate analyses are needed to determine how parallel divergence has happened, which is an important step in understanding the factors that drive evolutionary divergence and diversification (Langerhans and DeWitt 2004; Oke et al. 2017; Stuart et al. 2017).

In this study, we analyzed a comprehensive dataset, both in terms of genetic markers (∼20,000 SNPs) and geometric morphometric data, of almost 700 individuals covering nearly all lake populations and all described species of Midas cichlids. Complementing a previously in‐house generated RADseq dataset with new data for two more populations, we analyzed all data on Midas cichlids together in a complete phylogenetic framework of this entire lineage. This allowed us to infer the evolutionary relationships, test for introgression, and to compare the colonization histories of virtually all lineages in the entire species complex. Moreover, we generated geometric morphometric data for virtually all samples in our RADseq dataset to quantify the amount of body shape variation within lakes, as well as to determine the direction and extent of morphological divergence (length and angle of phenotypic trajectories) between crater lakes and their respective source population (i.e., by explicitly taking ancestor‐descendant relationships into account). Together with physico‐ecological attributes of the crater lakes (mean depth and size of the littoral zone), we then tested if the most relevant inferred demographic parameters (time since colonization, size of the founder population, admixture proportion from a secondary wave of colonization, and long‐term effective population size) can explain morphological divergence in Midas cichlids and if the rate of morphological change generally seems to remain constant, increase, or slow‐down with time after the colonization of a crater lake.

## Methods

### SAMPLING AND DDRAD SEQUENCING

Fish were collected in the field in 2001, 2003, 2005, 2007, 2010, and 2012 with gill nets or by harpooning. Collections of the great lakes were augmented with fish purchased from local fishermen (see Table S1 for sample sizes by population and location). Specimens were photographed in a standardized way from the lateral view in the field and tissue samples from fins or dorsal musculature were taken and preserved in pure ethanol. Whole genomic high‐molecular‐weight DNA was extracted with commercial kits (QiaGen DNeasy blood and tissue kit). Sequence data were generated using a double‐digest RAD sequencing approach (Peterson et al. 2012) with modifications as described previously (Recknagel et al. 2013a; Kautt et al. 2016a). To minimize the potential effect of PCR errors/duplicates, a low number of only 10 amplification cycles, 10 independent PCR reactions per library that were subsequently pooled, and a high‐fidelity polymerase were used for genomic library preparation. Reads were mapped to a genome assembly of *A. citrinellus* from Lake Nicaragua (Elmer et al. 2014) with *bwa mem v.0.7.12* (Li and Durbin 2009). Genotypes were called with *Stacks v.1.29* (Catchen et al. 2011; Catchen et al. 2013). On average 72,409 ± 17,189 (SD) RAD‐tag loci were obtained per individual with a mean coverage of 13.5 x ± 4.2 (SD). For details on data analysis and filtering see Kautt et al. (2016a).

### POPULATION STRUCTURE, ADMIXTURE AMONG CRATER LAKES, AND DIFFERENTIATION

Population structure and evolutionary relationships were investigated with *Admixture v.1.23* (Alexander et al. 2009), principal component analyses (PCA) using *Eigensoft v.5.0.2* (Patterson et al. 2006), and individual‐based phylogenetic split networks built with *SplitsTree v.4.13.1* (Huson and Bryant 2006). D‐statistics were calculated with qpDstat implemented in *AdmixTools v4.1* (Patterson et al. 2012) for all quadruplets of the form (((crater lake population, its source population), any other population), outgroup). Five individuals of a closely related Neotropical cichlids species, *Archocentrus centrarchus*, were used as outgroup for these analyses. Genetic differentiation (overall F_ST_) among populations was calculated with *Arlequin v3.5.1.3* (Excoffier and Lischer 2010) and statistical significance was assessed based on 10,000 permutations. Only loci with genotypes present in at least six samples (three in the case of the outgroup) per lake population and sympatric species therein and only a single SNP per RAD‐tag locus was used for all analyses above. No minor allele frequency filter was applied.

### DEMOGRAPHIC INFERENCE

The colonization history of crater Lake As. León was inferred using *fastsimcoal v.2.5.2.3* (Excoffier et al. 2013) using the same procedure and set of two‐population models as used previously for L. Apoyo and L. Xiloá (Kautt et al. 2016a), L. Masaya and L. Apoyeque (Machado‐Schiaffino et al. 2017), and L. As. Managua (Kautt et al. 2016b). We used *A. citrinellus* from L. Managua as a source population. Note, that the two species in the source lakes are genetically almost not differentiated (Table S2) and their site frequency spectra (SFS) almost identical (Fig. S1). Thus, choosing one or the other species (or both) as source population is unlikely to affect any of the analyses based on the SFS performed here. The source and crater lake populations were projected down to 50 and 30 alleles (25 and 15 individuals), respectively, to account for missing data (Gutenkunst et al. 2009). Genetic markers residing in coding regions were conservatively removed to minimize the potential effect of selection. Nonetheless, we note, that neutrality of markers is an assumption that is probably violated in certain cases. Reliably identifying and removing markers under selection in the bottlenecked crater lake populations is difficult, though, and could not be performed (Poh et al. 2014). By using genome‐wide markers, the effect of selection on certain markers, should be diluted, but we note that future inferences of the demographic history in Midas cichlids would benefit from using joint estimators of selection and demography once these methods are mature and generally applicable (Li et al. 2012; Bank et al. 2014). A detailed description of the methodological approach is provided in Kautt et al. (2016a). Long‐term average effective population sizes were inferred from models without population size changes.

### GEOMETRIC MORPHOMETRICS

Body shape was quantified for virtually all samples included in the RADseq dataset (Table S1) by digitizing 12 homologous landmarks in individual photographs taken from the lateral side of the fish with *TPSDIG 2.17* (Rohlf 2001). All landmarks were set by the same individual observer for this study to avoid interobserver bias. In order to make the data comparable to previous studies, the landmarks comprised a subset (landmarks 1, 5, 6, 7, 8, 9, 10, 11, 12, 13, 14, 15) of previously defined and used positions (Elmer et al. 2010a). Body shape divergence in form of Procrustes and Mahalanobis distances, and body shape variation were determined in *MorphoJ 1.06d* (Klingenberg 2011). Briefly, following Procrustes superimposition, a multivariate pooled within‐group regression of Procrustes coordinates on centroid size was performed. The regression residuals were then used to determine distances in order to correct for allometric effects. Pairwise morphological distances were calculated among lakes (i.e., individuals grouped by lake of origin). While distances were calculated based on a single global Procrustes superimposition, the variation in body shape per lake population was determined as the mean squared distance of individual landmarks to the centroids in separate lake‐specific Procrustes superimpositions. The coefficient of variation of the elongation index (CV_ei_) was calculated from two inter‐landmark distances that correspond to standard length and body height (Recknagel et al. 2014).

Individual and between‐group body shape variation was explored with principal component analyses (PCAs) and the *geomorph v.3.0.5* R package (Adams and Otarola‐Castillo 2013), again based on allometry‐corrected shape coordinates following a generalized Procrustes analysis. Since all crater lakes except for L. Apoyo were colonized from the same source lake of L. Managua (Barluenga and Meyer 2010; Machado‐Schiaffino et al. 2017), our data is not factorial and traditional phenotypic trajectory analyses (Adams and Collyer 2009) could not be performed. Instead, to visualize phenotypic trajectories, between‐group PCAs were performed and the corresponding least‐squares group means were connected (i.e. crater lake populations/species with their respective source population). The difference in length “dL” and angle “theta” of pairwise vectors in multivariate space, an approach originally pioneered by Adams and Collyer (2009), was formally quantified following Stuart et al. (2017). Briefly, dL is the sum of t‐statistics comparing the x and y coordinates (Procrustes‐superimposed and allometry‐corrected) of all 12 landmarks between two groups. Theta is the arccosine of the correlation coefficient of the t‐statistics. Statistical significance was determined based on 1000 label‐switch permutations (see Stuart et al. 2017 for details). Note that we took the absolute value of observed and permuted dL, which could otherwise be positive or negative depending on which population pair is listed first. For practical reasons (i.e., creating a paired design), data for the source population of Managua were duplicated several times in the pairwise vector analyses. Note that these pairwise tests were solely based on (allometry‐corrected) raw shape coordinates and should therefore not be affected by duplication of data (unlike tests based on principal component scores, for example). Based on a morphological argument, for the two crater lakes that contain thick‐lipped fish, L. Masaya and L. Apoyeque, both the thin‐lipped *A. citrinellus* and the thick‐lipped *A. labiatus* were used together as source population, whereas for the other crater lakes that do not contain thick‐lipped fish only *A. citrinellus* was used for the main analyses. To test the effect of this assumption, trajectory analyses were, however, repeated using either only *A. citrinellus* or both species (*A. citrinellus* and *A. labiatus*) together as source population for all crater lakes.

### REGRESSIONS OF DEMOGRAPHIC AND PHYSICO‐ECOLOGICAL VARIABLES ON MORPHOLOGICAL VARIABLES

All statistical analyses were performed in *R 3.1.2* (R Development Core Team 2014). In total, four different response variables were investigated: Overall body shape change of a crater lake and its respective source population in terms of Procrustes distance, variance in body shape of a crater lake, the coefficient of variation in body elongation (“CV_ei_”) (Recknagel et al. 2014), and the rate of morphological change per generation, defined as the Mahalanobis distance of a crater lake and its source divided by colonization time. Note that Mahalanobis distances scale by within‐group variance and are often regarded as a multivariate analog of the haldane (Lerman 1965; Gingerich 2009; Arnegard et al. 2010).

Six explanatory variables were tested: the four demographic variables colonization time in number of generations, size of the founder population in number of individuals, size of the admixture event (secondary wave of colonization from the source population) in proportion of gene pool that has been replaced, and long‐term effective population size in number of individuals, as well as the two eco‐physiological variables mean depth of the lake in meters and size of the littoral zone in km^2^. The latter two were obtained from Waid et al. (1999) and references therein. Another potentially informative variable, surface area (Wagner et al. 2012), was highly correlated with mean depth (*P* = 0.018, *R* ^2^ = 0.89) and thus omitted. We restricted the regression analyses to only four of the inferred demographic variables, since we deemed them among the most important factors determining the extent and rate of morphological evolution. We note, however, that the effect of admixture events is difficult to quantify in simple regression models, since the impact of genetic exchange depends likely on the amount and timing in a complex way. In other words, a large admixture event only few generations after the initial colonization would probably have less impact than a much smaller admixture event after a longer time of separation. Regressions were performed separately, because testing the effect of several explanatory variables and possible interactions together in one model was not sensible due to the low number of observations (six crater lakes).

## Results

### POPULATION STRUCTURE AMONG AND WITHIN LAKES

**Figure 2:**
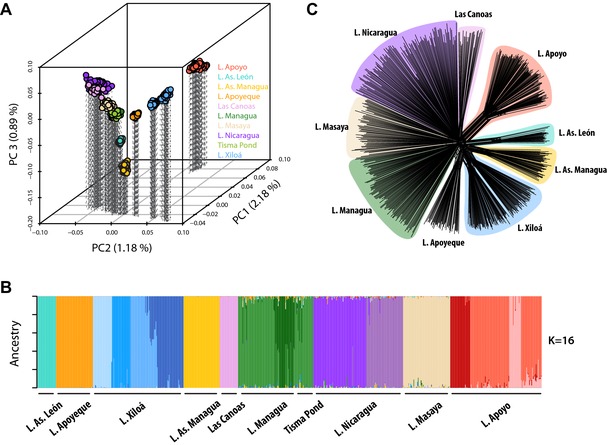
Overall genetic divergence based on 19,064 SNPs. (A) Individual variation along the first three axes of a principal component analysis (PCA) of genetic variation. All axes are highly significant. Note that the order of PC1 and PC2 is reversed for better visual representation of separation along PC2. (B) Individual Bayesian cluster assignment (*Admixture*) assuming *K* = 16 genetic clusters. (C) Neighbor‐net split graph based on genetic distance.

In agreement with their geographic isolation, each lake population formed a distinct genetic cluster, except for Tisma Pond, which was indistinguishable from L. Managua. (Fig. 2A, Fig. S2). An *Admixture* clustering analysis found the most supported number of genetic clusters (K) in our dataset to be between 14 and 16 (Fig. S3B), which additionally revealed some of the sympatric species within lakes (Fig. 2B). Assuming *K* = 16, the two species *A. citrinellus* and *A. labiatus* could be distinguished in both great lakes. Interestingly, despite the impressive dimensions of the great lakes—the maximum distance between our sampling localities in L. Nicaragua was approximately 150 km—we did not detect any grouping of individuals with regard to sampling locations within either great lake (Fig. S4). On the other hand, in the much smaller crater lakes Xiloá (∼2 km in diameter) and Apoyo (∼6.6 km), four (corresponding to *A. amarillo*, *A. viridis*, *A. sagittae*, and *A. xiloaensis*) and three genetic clusters (corresponding to *A. zaliosus* and two clusters of benthic individuals) were apparent, respectively. We note that a more fine‐scale population structure within the crater lakes has been addressed in detail before (Kautt et al. 2016a; Machado‐Schiaffino et al. 2017) and was thus not further investigated here.

### EVOLUTIONARY RELATIONSHIPS AND ADMIXTURE AMONG CRATER LAKES

An individual‐based phylogenetic split graph revealed that all lake populations were connected to the main central network and no two crater lakes diverged from a shared branch (Fig. 2C). This is consistent with an independent colonization of all crater lakes from the great lakes and previous studies that provided evidence that all crater lakes were colonized from L. Managua except for L. Apoyo, which was colonized from L. Nicaragua (Barluenga and Meyer 2010; Machado‐Schiaffino et al. 2017). We refer readers to the results of our demographic analyses for information on the order of divergence events (see below and Table 1).

**Table 1:** Crater lake colonization histories

	N_founder_	N_current_	admix	MIG_crater→source_	MIG_source→crater_	T_admix_	T_col_
L. Apoyeque*	111	14,717	0.162			376	577
	(46–201)	(1483–32,992)	(0.083–0.224)			(292–472)	(427–772)
L. Apoyo†	263	6461–43,960	0.043			892	1678
	(128–738)	(0–48,938)	(0.009–0.093)			(859–1538)	(1234–2257)
L. As. Managua‡	32	19,460	0.323		8.95 × 10^–5^	507	797
	(0–71)	(5336–43,039)	(0.184–0.501)		(5.40 × 10^–5^ – 1.14 × 10^–4^)	(384–652)	(516–1284)
L. As. León	169	9091	0.119	3.15 × 10^–5^	1.70 × 10^–5^	737	1550
	(0–237)	(6299–13,535)	(0.074–0.160)	(1.12 × 10^–5^ – 4.78 × 10^–5^)	(0–2.71 × 10^–5^)	(507–901)	(1352–1798)
L. Masaya*	8614§	8614	0.210	6.06 × 10^–5^		244	1561
	(7799–9761)	(7799–9761)	(0.145–0.292)	(1.51 × 10^–5^ – 9.77 × 10^–5^)		(116–395)	(1391–1789)
L. Xiloá†	146	12,144 – 35,544	0.286		1.72 × 10^–5^	891	1318
	(37 – 557)	(0–49,894)	(0.107–0.433)		(0 – 8.60 × 10^–5^)	(767 – 1374)	(1198 – 2064)

^*^Data from Machado‐Schiaffino et al. 2017.

^†^Data from Kautt et al. 2016a.

^‡^Data from Kautt et al. 2016b.

^§^Best demographic model did not include a population size change in crater lake.

Shown are maximum‐likelihood parameter point estimates and 95% confidence intervals based on 25‐50 parametric bootstrap replicates. For each crater lake, given are the inferred size of the founder population “Nfounder” and the current population size “Ncurrent” (a range in case of several sympatric species) in number of individuals, the proportion of admixture “admix” (i.e., secondary wave of colonization) from the source, migration rates “MIG” in proportion of alleles per generations (direction in forward time), and times of admixture event “Tadmix” and colonization “Tcol” in number of generations. Note that estimates specific to the source populations were omitted to enhance readability.

The independent histories of the crater lakes were also consistent with formal tests of admixture in form of D‐statistics (Patterson et al. 2012), with one exception: While almost all tests, comprising a crater lake forming a clade together with its source population versus any other nonsympatric population and an outgroup (*Archocentrus centrarchus*), were nonsignificant (Table S3), all four‐population tests involving a species from L. Xiloá and L. Apoyeque were significant (*P* < 0.01), providing evidence for gene flow between the two of them. Given the age and colonization history of the lakes, we believe migration from L. Xiloá into L. Apoyeque to be more likely than vice versa, but in the absence of suitable populations for five‐population tests (Eaton and Ree 2013; Pease and Hahn 2015) the direction of gene flow remains to be determined.

We note that defining a simple four‐population species tree in our system, in which multiple lakes have been colonized from the same source population, is nontrivial and the results should be considered with caution. Moreover, we note that these tests are not expected to be sensitive to admixture (secondary waves of colonization) from the same source population into a crater lake population (see below).

### COLONIZATION HISTORY

All crater lakes’ colonization histories were inferred using coalescent simulations and the site frequency spectrum (Excoffier et al. 2013). Note, that Las Canoas is not a crater lake, but a small lake that had been permanently connected by a riverine connection to L. Nicaragua until the construction of a dam. Thus, it was not included in these or subsequent analyses, but mainly included in the genetic analyses above, to rule out that it has contributed to the gene pool of any of the crater lakes (i.e., to test for introgression). Combining our results here with those of previous studies (Kautt et al. 2016a; Kautt et al. 2016b; Machado‐Schiaffino et al. 2017) allowed us to evaluate the demographic history of the Midas cichlid species complex in its entirety (Table 1). In the case of all crater lakes a very similar demographic model was most supported (for details see Kautt et al. 2016b). The colonization of the crater lakes happened recently (only between 580 and 1680 generations ago) and by a small founder population (ca. 30–260 individuals), which started to grow exponentially immediately after the colonization of the new lakes. Only in the case of L. Masaya was the improvement of the model gained by adding population growth outweighed by the penalty of adding an extra parameter (Anderson 2008). Thus, the best model for L. Masaya did not include a population size change. Apart from that, the models for all lakes were identical, differing only in the presence or absence of one or both of the possible migration rates between a crater lake and its source population (Table 1). Surprisingly, an admixture event from the source populations into the crater lakes was supported in all cases, albeit to varying degrees with a range of 4.3% in L. Apoyo up to 32.3% replacement of the resident gene pool in L. As. Managua.

While we consider the above described models as the best models, for L. As. León and L. Masaya a model in which the colonization happened before the bottleneck in the source lakes received higher support. Yet, these models involved divergence times that are older than the geologically determined ages of the crater lakes themselves (based on the assumption of a generation time of more than one year) and levels of genetic exchange that appear unrealistically high for remote crater lakes (relatively high bidirectional continuous gene flow together with admixture events on the order of 32.9 and 57.1%). Therefore, we deemed these models biologically unrealistic. Nonetheless, we present the estimates for both models for these two crater lakes in addition to what we consider the best models (Table S4) and performed regressions on morphological parameters also with these alternative demographic parameter estimates (Fig. S5).

The average long‐term effective population sizes (Table S5) of the source populations (around 20,000 individuals) were, as expected, an order of magnitude larger than the crater lake populations (ca. 1000–3000). Crater Lake Masaya was again an exception and, while not as genetically diverse as the source populations, its long‐term effective population size (ca. 8500) was estimated to be several times larger than that of any other single crater lake population.

### MORPHOLOGICAL DIVERGENCE, DIVERSITY, AND PARALLEL EVOLUTION

**Figure 3:**
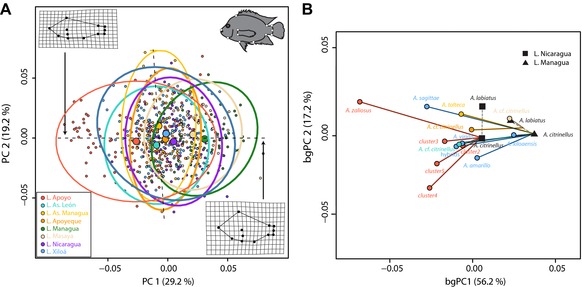
Overall morphological divergence based on 12 geometric morphometric landmarks. (A) Individual variation in body shapes along the first two axes of a principal component analysis (PCA). The main change in body shapes along PC1 is indicated by wireframe deformation grids. The positions of used landmarks are indicated on the top right. (B) Phenotypic trajectories along the first two axes of variation in a between‐group PCA (bgPCA) of populations’ least‐square means. Note that several crater lakes were colonized from the same source lake. In the case of crater lakes Apoyeque and Masaya trajectories originate from a hypothetical intermediate morphology of the two species in the source lakes, since these two lakes have likely been colonized by a mix of both of them.

The largest amount of variation in body shapes (principal component 1 with 29.2 %) was captured in overall body elongation (Fig. 3A). Strikingly, every single crater lake population was found to be generally more elongated than its respective source population and this phenotypic axis explained 56.2% of variation in a between‐group PCA (Fig. 3B). Interestingly, even within the two crater lakes that harbor each one formally described limnetic and several benthic species (L. Apoyo and L. Xiloá), all species–also the benthic ones—are at least slightly more elongated than the source populations (Fig. 3B). The two limnetic species, *A. zaliosus* in L. Apoyo and *A. sagittae* in L. Xiloá were clearly the most diverged species (Table 3) and have done so in a remarkable parallel fashion (Fig. 3B).

However, there were also significant differences in the extent and direction of crater lake divergence in multivariate space. Note that pairwise tests were based on all 24 allometry‐corrected landmark coordinates, whereas divergence was visualized along only the first two axes of a PCA. Midas cichlids in Crater Lake Masaya were significantly less diverged from their source population than almost all other crater lake populations, while the two limnetic species, *A. zaliosus* and *A. sagittae*, were significantly more diverged from their source population than any of the other populations (Table 3). Regarding the direction of change, 81 out of 91 pairwise comparisons were significantly different (Table 3). While most vectors were nonetheless more parallel than orthogonal (Table 3), the divergence vectors of four out of five species from L. Apoyo were almost orthogonal to L. Masaya (72.0–90.3°) and deviated thus from parallelism (Table 3). Assuming that all crater lakes were colonized by *A. citrinellus* only or by both species (*A. citrinellus* and *A. labiatus*) from the source lakes together did not qualitatively change these results (Table S6). In conclusion, all crater lake populations have mainly diverged in a parallel fashion by evolving overall more elongated body shapes, yet we also detected significant differences in divergence vectors that are related to other axes of body shape variation.

**Figure 4:**
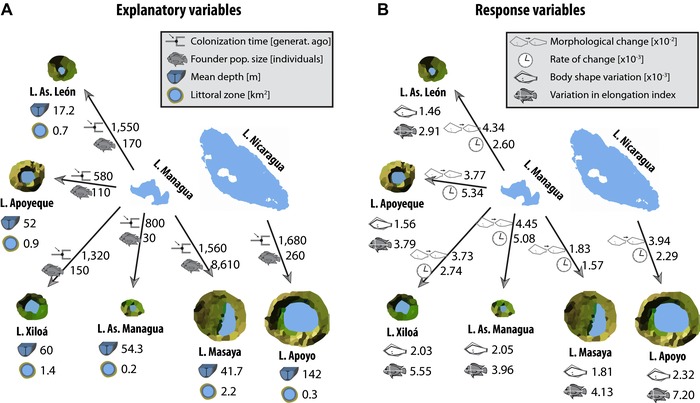
Schematic illustration summarizing main data used for regression analyses. (A) The four main explanatory demographic and ecological variables and (B) the four morphological response variables. Demographic parameters were inferred from genetic data here and in previous studies. Ecological/physical parameters were obtained from Waid *et al*. (1999). Morphological data were generated in this study. Morphological change = Procrustes distance, Rate of change = Mahalanobis distance/colonization time in generations. Note that only explanatory variables that were significantly correlated with at least one of the response variables are shown. See main text for details and Table S7 for all regression results.

In addition to morphological divergence, we were interested in the overall amount of body shape variation within crater lake populations. We found the highest variance in L. Apoyo, followed by, L. As. Managua, L. Xiloá, L. Masaya, L. Apoyeque, and L. As. León (Fig. 4B). A similar pattern (except that L. Apoyo was followed by L. Xiloá and L. Masaya) was obtained with a univariate measure of elongation, the coefficient of variation in elongation index (CV_ei_), supporting again the notion that body elongation is a key component in body shape variation in Midas cichlids.

### ASSOCIATION BETWEEN DEMOGRAPHIC AND ECOLOGICAL FACTORS AND MORPHOLOGY

**Figure 5:**
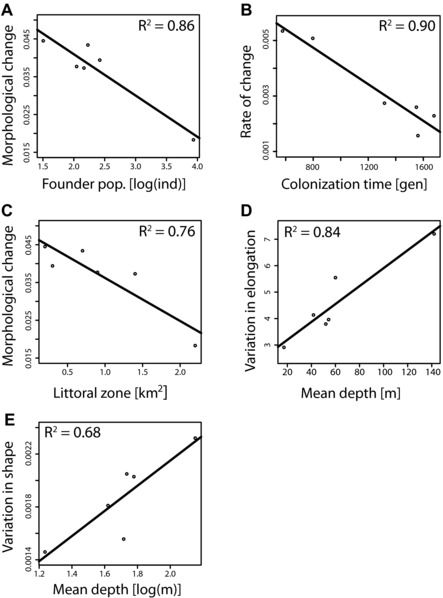
Linear regression analyses of demographic and ecological explanatory variables against morphological response variables. (A) Morphological divergence of a crater lake population compared to its source population is negatively associated with the size of the founder population. (B) The rate of this change decreases with the time since colonization. (C) Additionally, the extent of morphological change is negatively associated to the size of a crater lake's littoral zone. The variation of a crater lake population in terms of (D) body elongation and (E) overall body shape is positively associated with the mean depth of the respective crater lake. Shown are only significant regressions (*P* < 0.05). See Table S7 for all regression results. Note the log‐transformation of explanatory variables in some regressions.

The extent of morphological divergence of a crater lake population and its source was negatively correlated with the size of the founder population (adjusted *R* ^2^ = 0.861, *P* = 0.005) (Fig. 5A), but was not significantly correlated with time since colonization (Table S7). Note that founder population size was log‐transformed due to the large range of data points, spanning two orders of magnitude. But, this pattern was strongly driven by L. Masaya, which is a clear outlier in our dataset, since our best demographic model did not include a population size change and, therefore, the founder population size of L. Masaya equals the current effective population size. This is certainly unrealistic. We note, however, that if we use the alternative model of L. Masaya's colonization history (and consequently also the alternative model for L. As. León)–which included a population size change–the correlation was still significant and, in fact, became even stronger (adjusted *R* ^2^ = 0.893, *P* = 0.003) (Fig. S5A). Thus, our data showed a negative correlation between the extent of morphological divergence and founder population size, albeit this relationship is arguably strongly influenced by L. Masaya and has to be considered with caution. Neither the proportion of admixture (secondary colonization from the source lakes), nor the long‐term effective population sizes was significantly correlated with any of the morphological response variables.

Independent of which models’ (best or alternative) parameter estimates were used, the rate of morphological divergence decreased significantly with time (adjusted *R* ^2^ = 0.903, *P* = 0.002 for best models and adjusted *R* ^2^ = 0.964, *P* = 3.1 × 10^−4^ for alternative models) (Fig. 5B and Fig. S5B). In case of the alternative demographic models’ estimates an exponential relationship provided a much better fit to the data (adjusted *R* ^2^ = 0.964; Fig. S5B) than a linear one (adjusted *R* ^2^ = 0.718, *P* = 0.021), whereas for the best demographic models’ estimates a linear and exponential relationship fit almost equally well (*R* ^2^ = 0.903 and 0.894 with *P* = 0.002 and 0.003, respectively). Whether linearly or exponentially, these results suggest that body shape divergence progresses rapidly after the colonization of a crater lake and starts to slow down with time soon afterwards.

Concerning ecological factors, the extent (not rate) of morphological divergence of a crater lake population compared to its source population was negatively correlated with the size of the littoral zone (adjusted *R* ^2^ 0.758, *P* = 0.015) (Fig. 5C). Given that the source lakes represent an almost exclusively littoral habitat this association is in agreement with the notion that a larger change in habitat will result in a larger morphological change. Finally, our data not only confirm previous findings that the average depth of a crater lake predicts the amount of variation in body elongation (Recknagel et al. 2014) of its habitant population (adjusted *R* ^2^ = 0.843, *P* = 0.006) (Fig. 5D), but furthermore show that the overall variation in body shape is positively correlated with the mean depth of a crater lake (Fig. 5E). In the latter case, an exponential relationship (log‐transformed depth; adjusted *R* ^2^ = 0.675, *P* = 0.028) provided a better a fit to the data than a linear relationship (*R* ^2^ = 0.605, *P* = 0.042).

In summary, the extent of morphological change after the colonization of a crater lake seems to be larger in populations that were founded by fewer individuals and more pronounced in crater lakes that are more dissimilar compared to the source lake. The rate of this change slows down with time. The amount of body shape variation within a crater lake, especially body elongation, is positively associated with the average depth of a crater lake.

## Discussion

Midas cichlids in Nicaragua form a young radiation of closely related lineages inhabiting crater lakes that all stem from the same extant source populations in the great lakes (Barluenga and Meyer 2010). Which of these lineages constitute species is in our opinion subject to taxonomical issues (discussed in Text S1), but should not be of relevance to the main conclusions we have drawn here. Unlike other cichlids in these crater lakes (Fruciano et al. 2016), Midas cichlids have morphologically diverged from the source populations and in at least two crater lakes they have diversified in situ (Barluenga et al. 2006; Kautt et al. 2016a). Why and how (parallel) Midas cichlids diverge and when exactly they diversify were still largely unanswered questions. In this study, we aimed to test whether intrinsic (e.g., adaptive introgression, amount of genetic variation, time since colonization) or extrinsic factors (e.g., ecological opportunity) are better predictors of morphological divergence in Midas cichlids. We found evidence for introgression from L. Xiloá into L. Apoyeque (Table 2), and unexpectedly, our demographic analyses provided support for secondary waves of colonization from the source population into all crater lakes. While these admixture events may have facilitated diversification in certain cases (Kautt et al. 2016a), their effect is likely to be complex and not readily predictable (see below). After colonization, all crater lake populations have diverged from the source population in body morphology and the main direction is toward more slender body shapes. The extent of this divergence is best predicted by how dissimilar a crater lake is to its source population (size of littoral zone). The amount of body shape variation within a crater lake is best predicted by the mean depth of the lake. Time since colonization was not significantly associated with either divergence or amount of variation, but rather, the rate of morphological change is high in the beginning and seems to slow down with time. While the size of the founder population was (negatively) associated with phenotypic divergence, this result was driven by a single observation (L. Masaya) and we consider it with caution. Thus, we conclude that ecology is most likely the main factor explaining allopatric divergence and sympatric diversity in Midas cichlids.

**Table 2:** D‐statistics support a close genetic relationship between L. Apoyeque and L. Xiloá

Pop1 (W)	Pop2 (X)	Pop3 (Y)	Pop4 (Z)	D‐statistic	BABA	ABBA	Z‐score	*P*‐value
Aye_cit	Man_cit	Xil_sag	outgroup	0.1604	54	39	5.123	3.01 × 10^–7^
Aye_cit	Man_lab	Xil_sag	outgroup	0.1734	55	39	4.933	8.10 × 10^–7^
Aye_cit	Man_cit	Xil_hyb	outgroup	0.1539	52	38	4.849	1.24 × 10^–6^
Aye_cit	Man_lab	Xil_hyb	outgroup	0.1705	53	37	4.785	1.71 × 10^–6^
Aye_cit	Man_cit	Xil_xil	outgroup	0.1499	53	39	4.616	3.91 × 10^–6^
Aye_cit	Man_lab	Xil_xil	outgroup	0.1620	53	38	4.506	6.61 × 10^–6^
Xil_vir	Man_cit	Aye_cit	outgroup	0.1342	52	40	4.082	4.46 × 10^–5^
Aye_cit	Man_cit	Xil_vir	outgroup	0.1191	52	41	4.021	5.80 × 10^–5^
Xil_sag	Man_cit	Aye_cit	outgroup	0.1573	54	40	3.831	1.28 × 10^–4^
Aye_cit	Man_lab	Xil_vir	outgroup	0.1248	52	41	3.762	1.69 × 10^–4^
Xil_vir	Man_lab	Aye_cit	outgroup	0.1361	52	40	3.708	2.09 × 10^–4^
Xil_sag	Man_lab	Aye_cit	outgroup	0.1578	55	40	3.450	5.61 × 10^–4^
Xil_ama	Man_cit	Aye_cit	outgroup	0.1046	50	41	3.448	5.65 × 10^–4^
Aye_cit	Man_cit	Xil_ama	outgroup	0.1035	50	41	3.423	6.19 × 10^–4^
Aye_cit	Man_lab	Xil_ama	outgroup	0.1082	51	41	3.213	1.31 × 10^–3^
Xil_ama	Man_lab	Aye_cit	outgroup	0.1066	51	41	2.943	3.25 × 10^–4^
Xil_xil	Man_cit	Aye_cit	outgroup	0.1371	53	40	2.876	4.03 × 10^–3^
Xil_hyb	Man_cit	Aye_cit	outgroup	0.1258	52	40	2.837	4.55 × 10^–3^
Xil_xil	Man_lab	Aye_cit	outgroup	0.1397	53	40	2.771	5.59 × 10^–3^
Xil_hyb	Man_lab	Aye_cit	outgroup	0.1266	53	41	2.613	8.98 × 10^–3^

Populations are abbreviated by lake of origin (Aye = L. Apoyeque; Man = L. Managua; Xil = L. Xiloá) and species (cit = *A. citrinellus*; lab = *A. labiatus*; ama = *A. amarillo*; vir = *A. viridis*; hyb = hybrids; sag = *A. sagittae*; xil = *A. xiloaensis*).

Shown are all quadruplets of the form (((crater lake population W, source population X), any other nonsympatric population Y), outgroup Z) that returned a significant D‐statistic (*P* < 0.01). The complete list of performed tests is provided in Table S3.

### ISOLATION AFTER COLONIZATION VERSUS REPEATED COLONIZATIONS AND ADMIXTURE

Hybridization and genetic introgression can be important factors facilitating diversification (Abbott et al. 2013; Meier et al. 2017; Richards and Martin 2017) and one of our first objectives was to test for signs of genetic exchange among crater lakes. We found evidence for introgression among crater lakes in one case: most likely from L. Xiloá into L. Apoyeque. Given their close geographic proximity–the crater rims are only around 700 m apart–gene flow between these two lakes seems plausible. While this admixture event evidently did not trigger speciation, since L. Apoyeque only harbors a single population of Midas cichlids, it could have contributed to the overall morphological similarity of L. Xiloá and L. Apoyeque (Fig. 3, Table 3) and facilitated L. Apoyeque's population to evolve more slender body shapes as a response to the new selection pressure of the crater lake environment.

**Table 3:** Pairwise phenotypic divergence vector analyses

Difference in vector length “dL” in standard error units (*P*‐values above diagonal)															
Lake	**Species**	**AsL**	**AsM**	**Aye**	**Mas**	**Xil‐ama**	**Xil‐hyb**	**Xil‐sag**	**Xil‐vir**	**Xil‐xil**	**Apo‐cl2**	**Apo‐cl3**	**Apo‐cl4**	**Apo‐cl5**	**Apo‐zal**
L. As.León		*NA*	0.80	0.99	0.00	0.83	0.59	0.00	0.48	0.03	0.39	0.00	0.46	0.08	0.00
L. As. Managua		1.46	*NA*	0.61	0.00	0.90	0.76	0.01	0.85	0.01	0.03	0.00	0.39	0.02	0.00
L. Apoyeque		0.45	1.91	*NA*	0.00	0.97	0.97	0.00	0.81	0.16	0.15	0.00	0.81	0.22	0.00
L. Masaya		12.17	13.63	11.72	*NA*	0.00	0.06	0.00	0.00	0.00	0.01	0.36	0.00	0.02	0.00
L. Xiloá	***A. amarillo***	0.77	0.69	1.22	12.94	*NA*	0.46	0.00	0.54	0.01	0.31	0.00	0.34	0.02	0.00
	**hybrids**	4.28	5.75	3.84	7.88	5.06	*NA*	0.00	0.31	0.96	1.00	0.09	0.92	0.83	0.00
	***A. sagittae***	14.39	12.93	14.84	26.56	13.62	18.68	*NA*	0.00	0.00	0.00	0.00	0.00	0.00	0.00
	***A. viridis***	2.76	1.29	3.20	14.92	1.98	7.04	11.64	*NA*	0.00	0.13	0.00	0.17	0.00	0.00
	***A. xiloaensis***	5.65	7.11	5.20	6.52	6.42	1.36	20.04	8.40	*NA*	0.75	0.01	0.74	0.92	0.00
L. Apoyo	**cluster2**	4.51	5.97	4.06	7.66	5.28	0.23	18.90	7.26	1.14	*NA*	0.19	0.97	0.79	0.00
	**cluster3**	10.66	12.12	10.21	1.51	11.43	6.38	25.05	13.41	5.01	6.15	*NA*	0.07	0.17	0.00
	**cluster4**	3.60	5.06	3.15	8.57	4.37	0.69	17.99	6.35	2.05	0.91	7.06	*NA*	0.75	0.00
	**cluster5**	5.91	7.37	5.46	6.26	6.68	1.63	20.30	8.66	0.26	1.40	4.75	2.31	*NA*	0.00
	***A. zaliosus***	28.98	27.52	29.43	41.15	28.21	33.26	14.59	26.23	34.63	33.49	39.64	32.58	34.89	*NA*
Difference in vector angle “theta” in degrees (*P*‐values above diagonal)															
L. As.León		*NA*	0.02	0.47	0.00	0.00	0.09	0.00	0.01	0.00	0.00	0.00	0.03	0.00	0.00
L. As. Managua		29.16	*NA*	0.00	0.00	0.00	0.01	0.01	0.01	0.00	0.00	0.00	0.01	0.00	0.01
L. Apoyeque		18.45	31.11	*NA*	0.00	0.01	0.30	0.01	0.46	0.00	0.00	0.00	0.14	0.01	0.00
L. Masaya		55.23	50.82	50.36	*NA*	0.00	0.07	0.00	0.00	0.00	0.00	0.07	0.39	0.01	0.00
L. Xiloá	***A. amarillo***	35.64	41.94	31.11	62.74	*NA*	0.00	0.00	0.00	0.00	0.00	0.00	0.03	0.08	0.00
	**hybrids**	27.45	42.92	27.22	54.89	38.27	*NA*	0.00	0.02	0.01	0.00	0.01	0.01	0.00	0.00
	***A. sagittae***	30.08	27.56	27.50	48.87	48.59	37.01	*NA*	0.02	0.00	0.00	0.00	0.00	0.00	0.00
	***A. viridis***	26.59	28.36	18.34	51.74	29.91	32.09	22.17	*NA*	0.00	0.00	0.00	0.00	0.00	0.00
	***A. xiloaensis***	63.37	64.74	59.82	64.17	56.26	48.64	64.40	58.21	*NA*	0.00	0.00	0.00	0.00	0.00
L. Apoyo	**cluster2**	72.25	78.44	67.27	91.33†	58.38	68.64	74.77	64.92	78.61	*NA*	0.00	0.00	0.00	0.00
	**cluster3**	47.21	53.69	56.09	83.42†	53.97	50.54	62.34	59.93	70.99	63.60	*NA*	0.07	0.01	0.00
	**cluster4**	42.51	56.32	41.77	70.02	39.36	42.92	59.65	49.16	61.94	59.99	46.47	*NA*	0.34	0.00
	**cluster5**	43.83	57.18	41.71	74.20†	29.39	44.35	61.19	46.24	69.39	45.61	46.12	29.22	*NA*	0.00
	***A. zaliosus***	35.35	32.20	39.02	71.96†	50.99	42.74	34.55	39.94	67.49	65.95	41.83	56.67	55.69	*NA*

^†^Proportion of bootstrapped distribution that included 1.57 radians (90 degrees) multiplied by two for a two‐tailed approach was > 0.05. In all other pairwise comparisons proportion was below ≤ 0.05, indicating that direction of vectors is more parallel than orthogonal. See Stuart et al. (2017) for details.

Vector length was calculated as the sum of *t*‐statistics of all 24 *x*–*y* landmark coordinate comparisons of a crater lake and its source population. Vector angle was calculated as the arccosine of the correlation coefficient of these *t*‐statistics. See methods section for details. Statistical significance was assesses with 1000 permutations.

Apart from this one admixture event among crater lakes, we found support for an admixture event from the source population into the crater lake populations in all cases (i.e., a secondary wave of colonization). Except for L. Xiloá, which may have been connected to the closely located great L. Managua (Fig. 1) as recently as 2000 years ago due to pronounced water level fluctuations and the low crater rim of L. Xiloá (Cowan et al. 2002), all other crater lakes are very remote and have to the best of our knowledge never been connected to any other water body. Thus, the strong support for admixture events in all cases was unexpected, especially since we previously found more evidence for a single versus multiple colonizations events even for L. Xiloá (Elmer et al. 2013). We note, however, that the results of Elmer et al. (2013) were based on a single nonrecombining locus (mtDNA) and therefore of limited power compared to the genome‐wide data used in this study. Yet, in any case, fish must have somehow been transported into the crater lakes in the first place (e.g., by birds, humans, or hurricanes (Bajkov 1949; Elmer et al. 2013)) and it is certainly possible that such events have occurred repeatedly. Continuous gene flow, on the other hand, is hard to imagine; especially in the direction from the crater lakes into the great lakes.

From a technical point of view, distinguishing between recent divergence with little gene flow versus more ancient divergence with more gene flow, especially in populations that have undergone recent bottlenecks, remains challenging (Loh et al. 2013; Hey et al. 2015). Thus, we warrant caution concerning the presence and magnitude of the inferred admixture events and levels of continuous gene flow. If confirmed, our results would in any case suggest that admixture events probably have a rather complex influence on diversification rates: the crater lake with the highest species richness, L Apoyo, was inferred to have experienced the lowest proportion of admixture (Table 1). Lake As. León, on the other hand, is inhabited by the least variable population, although it shares a similar colonization and admixture history with L. Xiloá , which harbors four species. Therefore, we tentatively conclude that the exact effects of admixture events from the source populations will likely vary and depend on the timing, size, and exact allelic contribution in a complex way.

### SIMILARITIES AND DIFFERENCES AMONG CRATER LAKE POPULATIONS’ HISTORIES

The inferred colonization histories of the crater lake populations mostly conform to our expectations and involve a founding event by a few dozen to a few hundred fish, which started to grow exponentially after colonization. While the colonization histories of most crater lakes are very similar, all of our analyses suggest that Crater Lake Masaya has retained much more of the ancestral genetic variation stemming from the source lakes than any other crater lake population, as evidenced by its close genetic affiliation to the source lakes in the genetic PCA and split graph (Fig. 2), levels of genetic differentiation (Table S2), and inferred demographic history (Table 1). To our knowledge, L. Masaya has never been connected to the great lakes and its geographic location (elevation profile) makes it hard to imagine a historical riverine connection. Why and how L. Masaya exhibits such an unusual pattern remains unknown. Nonetheless, knowledge about the different demographic histories of the crater lake populations provides valuable information for future studies that, for example, aim to elucidate the genetic bases underlying adaptive traits in Midas cichlids (e.g., association studies, Rosenberg et al. 2010).

### FACTORS DRIVING ALLOPATRIC BODY SHAPE DIVERGENCE IN MIDAS CICHLIDS

The adaptive radiation of Midas cichlids has occurred (and is probably still ongoing) at two different hierarchical levels. At the first level, that is, among allopatric crater lake populations, we show that the main direction of morphological change was remarkably parallel (Fig. 3B). All crater lake populations, and even all sympatric species within crater lakes, evolved a more slender body shape than their source population. We note, that trajectories of sympatric species are not fully independent, but would ideally take the morphology of shared ancestors into account. Due to a lack of fossil records this was not possible and we cannot rule out that benthic species became secondarily more deep‐bodied again. Nonetheless, their current body shapes are still more elongated than the source population. Given the absence of fossil data, we can, however, also not rule out that the apparent parallelism of the crater lake populations is actually due to the evolution of deeper bodies in the source lakes. The assumption of evolutionary stasis (of body shapes) in the source lakes rests on the fact that the bathymetric profile of the huge and shallow source lakes has most likely not changed considerably since the colonization of the crater lakes a few thousand years ago. Moreover, the phenotypic spaces of the source lakes overlap largely with that of Crater Lake Masaya (Fig. 3A), supporting the notion that the presumed ancestral state has mostly persisted in this crater lake and rendering directional evolution in the source lakes unlikely. A change in the overall direction toward more slender body shapes in the crater lakes is also consistent with eco‐morphological considerations (Webb 1984; Langerhans and Reznick 2010); crater lakes provide more habitat in which free‐swimming is more important than maneuvering. Moreover, the repeated evolution of more slender body shapes in crater lakes has also been found in many populations of crater lake cichlids in Uganda (Machado‐Schiaffino et al. 2015).

While all crater lake populations evolved mostly in parallel toward more slender body shapes (which explained the majority of body shape variation), pairwise analyses revealed that almost all phenotypic trajectories occurred in significantly different directions (Table 3). This deviation from complete parallelism could be due to random processes or reflect local adaptation (Stuart et al. 2017). Future studies investigating additional phenotypic traits and other lake‐specific parameters (e.g., chemical, available diet) would be interesting and might reveal factors that explain the difference in divergence vectors (e.g., Stuart et al. 2017). In any case, our result here is consistent with a previous study that found all populations to be morphologically distinct (Elmer et al. 2010a), and supports the notion that each allopatric crater lake population contributes to the overall phenotypic diversity of this species complex.

Apart from occurring mostly along a predicted direction, our data suggest that the *extent* of divergence is to some extent predictable: divergence is more pronounced in crater lakes that have a smaller littoral zone and, therefore, presumably present a more dissimilar environment from that of the source lakes. This independently evolved fit between ecology and morphology both in direction and extent provides strong support for the role of natural selection in shaping body shape evolution (Losos et al. 1998; Nosil et al. 2002). Interestingly, our results also show that the rate of morphological change decreases with time since colonization, suggesting that the pace of morphological change is very rapid shortly after the colonization of a crater lake and then slows down with time. This finding is in agreement with the potentially analogous rapid morphological changes of mammals after the colonization of islands (Millien 2006) and the more general pattern that evolutionary rates are not maintained over longer time scales (Kinnison and Hendry 2001; Hendry 2017, pp. 71–75). Furthermore, it confirms the theoretical prediction that bursts of rapid diversification rather than constant rates throughout time are expected in adaptive radiations (Gavrilets and Losos 2009; but see Harmon et al. 2010) and that adaptation to a new environment can happen extremely rapidly (Losos et al. 1997; Reznick et al. 1997; Lescak et al. 2015).

Interestingly, considering the speed of morphological change and the detected founder events, our results imply that Midas cichlids were not hindered in their ability to respond quickly to the new selection pressures of the crater lake environment, despite an apparent reduction in genetic variation. We note that body shape seems to be to a large extent genetically determined in Midas cichlids, as evidenced by the maintenance of distinct body shapes in the laboratory (Franchini et al. 2014) and a failure of plasticity experiments to induce more elongated body shapes (Kautt et al. 2016b).

Adaptation to a novel environment is usually expected to be faster when large amounts of standing genetic variation are available (Barrett and Schluter 2008; Reid et al. 2016). On the contrary, we found that morphological divergence was larger in crater lake populations that were founded by fewer individuals. This suggests that founder effects might facilitate morphological evolution, as envisioned by Mayr (1954). Empirical support for the effect of founder events has been shown in Anolis lizards (Kolbe et al. 2012). Yet, the correlation in our dataset was mainly driven by one crater lake (L. Masaya) and the robustness of this result is thus debatable. Generally, with only six crater lakes (observations) our statistical analyses are naturally limited. Multivariate models taking possible interactions among the explanatory variables into account and rigorous model testing approaches were therefore not sensible. Nonetheless, although they may be limited in number, studies of natural replicates are arguably the most meaningful way to identify and understand the actual mechanisms that drive evolution in the wild (Hendry 2017, p.3).

### FACTORS EXPLAINING THE PROPENSITY FOR SYMPATRIC DIVERSIFICATION

The second level that has contributed to the diversity of Midas cichlids is the diversification that has happened within crater lakes (Barluenga et al. 2006; Kautt et al. 2016a). The multiple endemic species inhabiting crater lakes Apoyo and Xiloá can be regarded as small‐scale adaptive radiation within the larger adaptive radiation of the entire species complex. But, stemming from the same source population, why did diversification take place in these two crater lakes and not the other ones? Our results show that the amount of morphological variation within a crater lake is positively correlated with the mean depth of a crater lake (Fig. 5D and E). This result quantitatively expands on Recknagel et al. (2014), who found the same correlation with a simple univariate measure of body elongation (“CV_EI_”). Neither the size of the founder population nor the time since colonization were significantly positively associated with the amount of body shape variation (Table S6). Thus, we think that ecological opportunity in the form of habitat diversity is the main factor that explains whether sympatric diversification will happen in Midas cichlids or not. The role of other fish species is difficult to quantify, but, qualitatively, the presence or absence of other fish does not seem to affect the propensity for sympatric diversification in Midas cichlids (see Text S2 for more details).

At a larger taxonomic scale, the questions of why exactly Midas cichlids are diversifying so rapidly remains open. In this respect, a closely related lineage of Midas cichlids, *Archocentrus centrarchus*, has also colonized several of the crater lakes, but not diverged morphologically in any way: unlike Midas cichlids, individuals of *A. centrarchus* from L. Xiloá are morphologically indistinguishable from their source in L. Managua and have not diversified into several genetic clusters in sympatry (Fruciano et al. 2016). Priority effects are unlikely to explain this pattern, as *A. centrarchus* and *Neetroplus nematopus*–another Neotropical cichlid—presumably share a very similar colonization history with Midas cichlids in L. Xiloá (Elmer et al. 2013; Franchini et al. 2017). Whether Midas cichlids exhibit any intrinsic features (e.g., genetic architecture of adaptive traits and mate choice) that make them more prone to diverge and diversify than other cichlids in Nicaragua is an ongoing research question that we are currently addressing with hundreds of completely sequenced genomes.

## Conclusions

Our results suggest that morphological diversity in the Midas cichlid species complex is to a large extent influenced by ecological factors in a deterministic way. This conclusion is in line with other studies of well‐known organismal radiations (Losos et al. 1997; reviewed in Schluter 2000), and further supports the preeminent role of natural selection in shaping biodiversity. Morphological diversification in Midas cichlids happens at two different hierarchical levels. After the colonization of a crater lake, Midas cichlids diverge morphologically most strongly, though not exclusively, from their source population toward more slender body shapes. In the case of one crater lake, L. Apoyeque, this might have been facilitated by introgression from L. Xiloá. Across the entire species complex, however, admixture among crater lakes or in the form of secondary waves of colonization from the same source cannot explain the pattern of body shape evolution. Instead, divergence is more pronounced in crater lakes that are more dissimilar compared to the source lakes. Interestingly, morphological divergence seems to happen rapidly after colonization, possibly associated with ecological divergence and speciation and decreases with time in all of the crater lake radiations. Finally, deeper crater lakes allow for a larger variation in body shapes of their resident population, which, in turn, is positively associated with the number of species such crater lakes can sustain. Overall, our results support a general scenario in which fish evolve toward a new adaptive optimum after the colonization of a crater lake by directional selection (i.e., adaptation to the crater lake environment in general) and then possibly start to diversify via disruptive (divergent) selection within a lake if ecological opportunities (e.g., deep crater lakes providing environmental heterogeneity) exist. Especially in the context of multispecies lakes (L. Apoyo and L. Xiloá) this is in good agreement with theoretical expectations of competitive speciation models (Rosenzweig 1978; Pimm 1979; Gavrilets 2004, p. 410). More generally, this study shows how an integration of molecular and morphological data in a young system of natural replicates–a rare and uniquely suited natural experiment—can help to further our understanding of how biodiversity is generated and why diversification rates differ among taxa; even closely related ones stemming from the same source population.

## DATA ACCESSIBILITY

Raw sequence data (fastq files) have been deposited on the European Nucleotide Archive (ENA) under Project‐Numbers PRJEB12689 (Kautt et al. 2016a) and PRJEB27345. Morphometric data (TPS file) is available as Data S1 in the supplementary information.

Associate Editor: Z. Gompert

## Supporting information


**Figure S1**

**Figure S2**

**Figure S3**

**Figure S4**

**Figure S5**

**Table S1**. Sample sizes by lake of origin and species for RADseq and geometric morphometrics.

**Table S2**. Pairwise levels of genetic differentiation in terms of F_ST_‐values.

**Table S3**. D‐statistics of all quadruplets comprising a crater lake population (W) and its source population (X) as a clade versus any other population (Y) and an outgroup (Z; *Archocentrus centrarchus)*. Analyses are based on 13526 SNPs.

supporting information

**Table S5**. Estimates of average long‐term effective population sizes.

**Table S6**. Pairwise phenotypic divergence vector analyses assuming that either both species in source lakes or *A.citrinellus* alone has colonized all crater lakes.

**Table S7**. Linear regression analyses between morphological response variables and demographic and physico‐ecological explanatory variables of six crater lake populations.

supporting Information

supporting Information

supporting Information
