# Supplementary figures and images for: Lessons from a natural experiment: Allopatric morphological divergence and sympatric diversification in the Midas cichlid species complex are largely influenced by ecology in a deterministic way
Source: Evol Lett. 2018 Jun 27;2(4):323–40. doi: 10.1002/evl3.64 (PMC6121794; doi:10.1002/evl3.64)

Two species within source lake

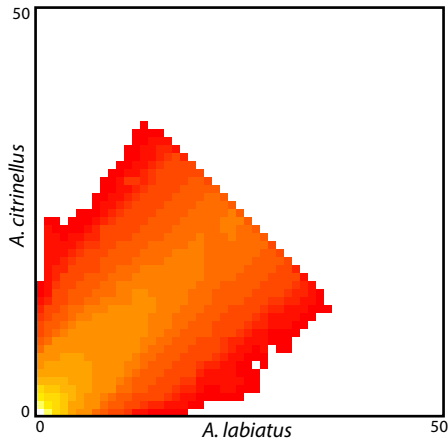

Source lake versus crater lake population

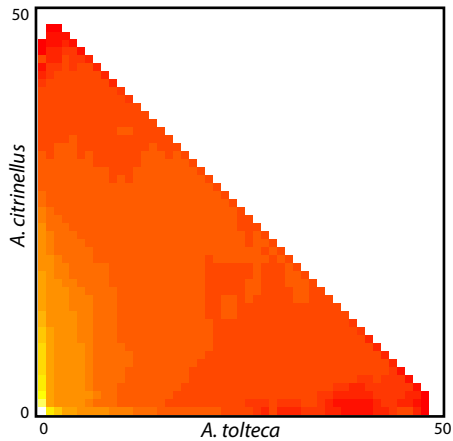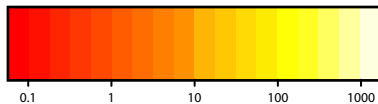

Supplement: Supplementary file 1 — Figure S1 [file EVL3-2-323-s001.pdf]

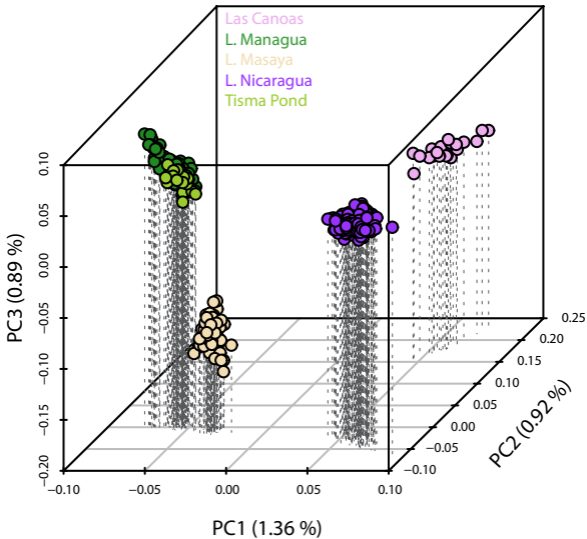

Supplement: Supplementary file 2 — Figure S2 [file EVL3-2-323-s002.pdf]

**A**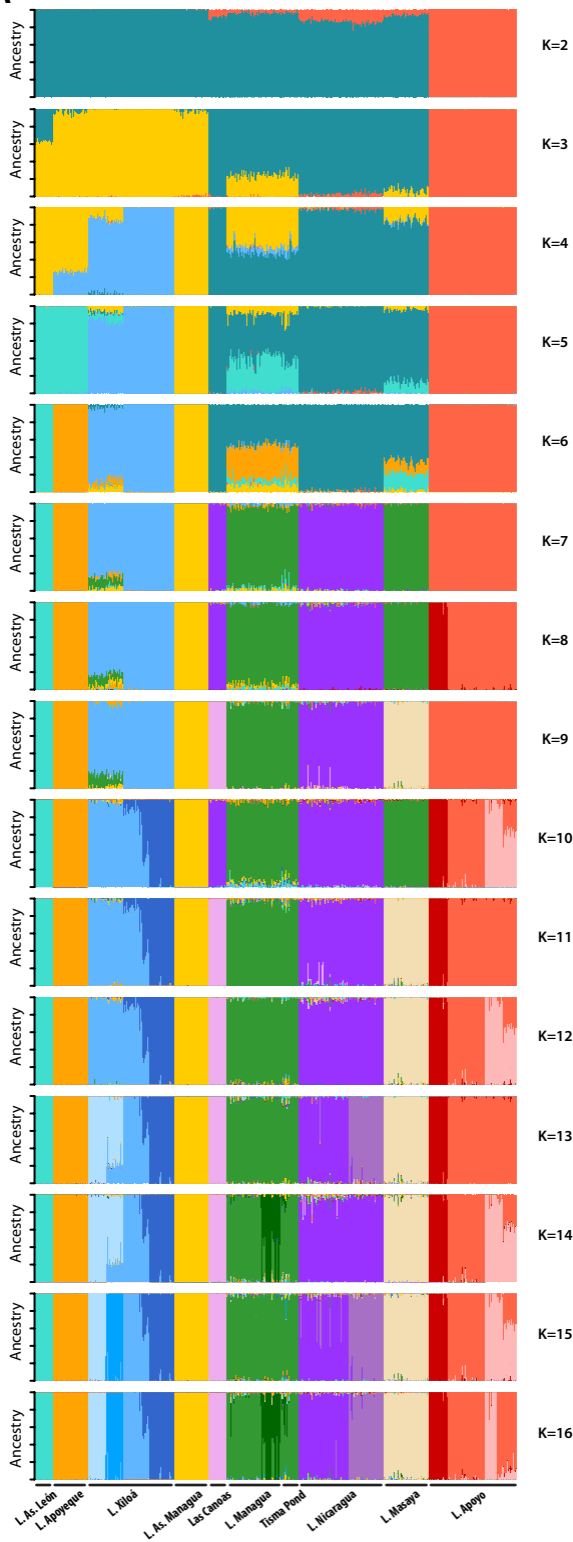**B**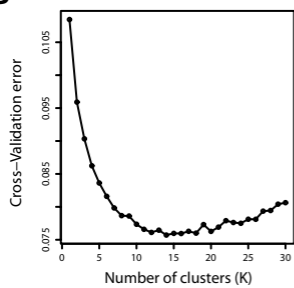

Supplement: Supplementary file 3 — Figure S3 [file EVL3-2-323-s003.pdf]

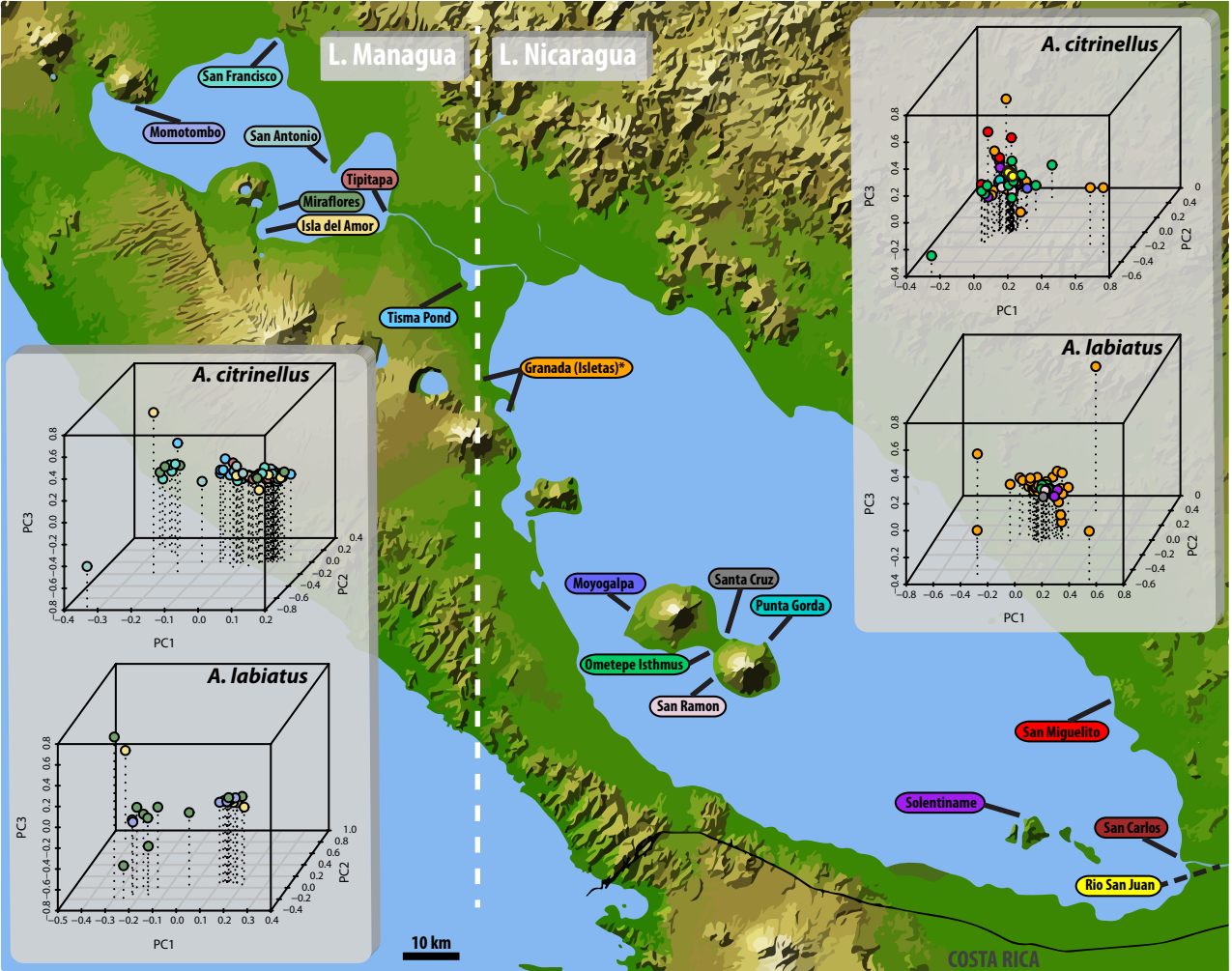

Supplement: Supplementary file 4 — Figure S4 [file EVL3-2-323-s004.pdf]

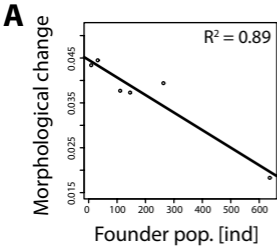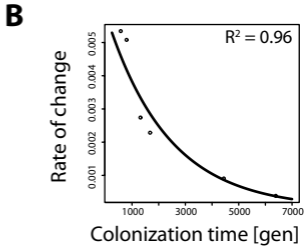

Supplement: Supplementary file 5 — Figure S5 [file EVL3-2-323-s005.pdf]
