# Supplementary material for: Lessons from a natural experiment: Allopatric morphological divergence and sympatric diversification in the Midas cichlid species complex are largely influenced by ecology in a deterministic way
Source: Evol Lett. 2018 Jun 27;2(4):323–40. doi: 10.1002/evl3.64 (PMC6121794; doi:10.1002/evl3.64)
Supplement: Supplementary file 13 — supporting Information [file EVL3-2-323-s013.docx]

**Taxonomic ambiguity and species concepts**

From a taxonomical point of view, Midas cichlids provide a clear example that species are more readily defined in sympatry than in allopatry (Coyne & Orr 2004). For example, the populations of the two species *A. citrinellus* and *A. labiatus* in the great lakes Nicaragua and Managua are genetically more closely related to their sympatric heterospecifics than to their allopatric conspecifics. In other words, *A. citrinellus* from L. Nicaragua is genetically more closely related to *A. labiatus* from L. Nicaragua than it is to *A. citrinellus* from L. Managua and *vice versa*. Despite the fact that mate choice is completely assortative in the laboratory (Machado-Schiaffino *et al.* 2017), we think the only plausible explanation for this pattern is the persistence of some gene flow between the two species after the two great lakes were separated. Thus, there is a conflict between the taxonomic species description, the genealogical species concept, and the biological species concept.

Similarly, it is debatable whether every single crater lake population should be described as a separate species. In the case of L. As. Managua the resident population was formally described as *A. tolteca* due to its genetic distinctness and morphological divergence compared to all other Midas cichlid species (Recknagel *et al.* 2013). Our data show, however, that the populations in L. As. León and L. Apoyeque are genetically and morphologically just as distinct. Thus, all crater lake populations can be considered genealogical species, although they may not constitute isolation/cohesion species (*sensu* Harrison 1998). While theory predicts that incompatibilities between isolated populations will evolve eventually (Gavrilets 2004), whether any intrinsic reproductive barriers among lake populations already exist remains to be tested.

In the sympatric settings of crater lakes Apoyo and Xiloá, which harbor each several described endemic species, the situation is less ambiguous. Individuals are grouped into genetically distinct clusters that are also mostly morphologically distinguishable (Table 2, Fig 3C). The absence of geographic barriers in these crater lakes implies that intrinsic reproductive barriers are keeping the species apart and at least at this (more progressed) stage of the speciation process laboratory experiments suggest that strong assortative mate choice presumably plays a predominant role (Baylis 1976; Kautt *et al*., unpublished data). However, as we have pointed out before, the current taxonomy in L. Apoyo is not in agreement with population genetic data and we only recognize five genetic clusters that mostly agree with five, but not six, of the described species (Kautt *et al.* 2016a). While *A. zaliosus* is unambiguous and *A. astorquii*, *A. chancho*, and *A. globosus* correspond predominantly to clusters 2-4, respectively, *A. flaveolus* and *A. supercilius* present the most difficulties and are mostly lumped into cluster 5 (Table S1).

Our results provide no support for geographic distance alone being a strong reproductive barrier in Midas cichlids. The two most distant sampling sites in our data set in L. Nicaragua are more than 150 kilometers apart. Despite this considerable distance we did not detect any genetic structuring by localities within the great lakes (Fig. S3). Hence, we conclude that Midas cichlids form essentially panmictic populations in the great lakes and individuals cover presumably large dispersal distances. In the crater lakes, which are several orders of magnitude smaller, geographic isolation alone is accordingly very unlikely to have influenced population divergence. This stands in contrast to many cichlid species in the East African Great lakes that are characterized by a high degree of philopatry and in which even small geographic distances are thought to contribute to speciation (Markert *et al.* 1999; Danley & Kocher 2001). We note, however, that this does not rule out that spatial isolation resulting from the differential use of habitats - such as the open-water zone versus the shore - facilitated divergence within the crater lakes. On the contrary, habitat isolation could have been, and possibly still is, an important factor contributing to the early stages of sympatric divergence in Midas cichlids (Kautt *et al.* 2016b).

In conclusion, the current thirteen-species taxonomy is not consistently based on a set of objective criteria. Hence, we advocate to adhere to an objective genetic cluster species complex (Mallet 1995). Future studies that test whether pre-mating or intrinsic postzygotic reproductive barriers exist among lake populations might help to reconcile the current taxonomic ambiguity.

**References**

Baylis, J. R. 1976. Quantitative Study of Long-Term Courtship: 1. Ethological Isolation between Sympatric Populations of the Midas Cichlid, *Cichlasoma citrinellum*, and the Arrow Cichlid, *C. zaliosum*. *Behaviour* 59:59-69.

Coyne, J. A., and H. A. Orr. 2004. *Speciation*. Sinauer Associates, Sunderland, Mass.

Danley, P. D., and T. D. Kocher. 2001. Speciation in rapidly diverging systems: lessons from Lake Malawi. *Molecular Ecology* 10:1075-1086.

Gavrilets, S. 2004. *Fitness landscapes and the origin of species*. Princeton University Press, Princeton, N.J.

Harrison, R. G. 1998. *The Relevance of Species Concpets for the Study of Speciation*. Pp. 19-31 *in* D. J. Howard, and S. H. Berlocher, eds. Endless Forms: species and speciation. Oxford University Press, New York.

Kautt, A. F., G. Machado-Schiaffino, and A. Meyer. 2016a. Multispecies Outcomes of Sympatric Speciation after Admixture with the Source Population in Two Radiations of Nicaraguan Crater Lake Cichlids. *PLoS Genetics* 12:

Kautt, A. F., G. Machado-Schiaffino, J. Torres-Dowdall, and A. Meyer. 2016b. Incipient sympatric speciation in Midas cichlid fish from the youngest and one of the smallest crater lakes in Nicaragua due to differential use of the benthic and limnetic habitats? *Ecology and Evolution* 6:5342-5357.

Machado-Schiaffino, G., A. F. Kautt, J. Torres-Dowdall, L. Baumgarten, F. Henning, and A. Meyer. 2017. Incipient speciation driven by hypertrophied lips in Midas cichlids fish? *Molecular Ecology*

Mallet, J. 1995. A Species Definition for the Modern Synthesis. *Trends in Ecology & Evolution* 10:294-299.

Markert, J. A., M. E. Arnegard, P. D. Danley, and T. D. Kocher. 1999. Biogeography and population genetics of the Lake Malawi cichlid Melanochromis auratus: habitat transience, philopatry and speciation. *Molecular Ecology* 8:1013-1026.

Recknagel, H., H. Kusche, K. R. Elmer, and A. Meyer. 2013. Two new endemic species in the Midas cichlid species complex from Nicaraguan crater lakes: *Amphilophus tolteca* and *Amphilophus viridis* (Perciformes, Cichlidae). *Aqua* 19:
