# Supplementary material for: Lessons from a natural experiment: Allopatric morphological divergence and sympatric diversification in the Midas cichlid species complex are largely influenced by ecology in a deterministic way
Source: Evol Lett. 2018 Jun 27;2(4):323–40. doi: 10.1002/evl3.64 (PMC6121794; doi:10.1002/evl3.64)
Supplement: Supplementary file 14 — supporting Information [file EVL3-2-323-s014.docx]

**Role of other fish species on propensity for sympatric diversification**

Competition and predation by other fish species (bird predators occur at all lakes) could be an important factor influencing ecological opportunity. Testing the (historical) role of other fish species for morphological evolution in Midas cichlids is, however, complicated for at least two reasons. First, simply using the occurrence of a potential competitor ignores the time axis and thereby potential priority effects. That being said, we have recently begun to investigate the colonization history of some non-Midas cichlid (*Archocentrus centrarchus* and *Neetroplus nematopus*) species in some crater lakes (L. Xiloá and L. Masaya) and found so far that they probably came into the crater lakes around the same time as Midas cichlids (Elmer et al. 2013; Franchini et al. 2017). A more comprehensive investigation of the colonization times of other fish species and all crater lakes will provide important baseline information about the potential for priority effects, but we believe this is a major endeavor and beyond the scope of this study. Second, and probably more importantly, it is very difficult to assign clear ecological roles to many of the other fish species. Midas cichlids are generalists and will feed on almost anything from Aufwuchs, algae, invertebrates, to small fish (Barlow 1976). Moreover, it is hard to predict if the presence of a competitor or predator is expected to increase or constrain morphological evolution in Midas cichlids. We summarize the presence of other fish species in all six crater lakes based on Elmer et al. (2010) and personal observations in the table below and tentatively assign them to ecological roles (potential prey, potential competitor, predator, unclear) based on their size and trophic niche (obtained from www.fishbase.org), but this is certainly an over-simplification.

| **Species** | **role** | **Apoyo** | **Masaya** | **As. León** | **Apoyeque** | **Xiloá** | **As. Managua** |
| --- | --- | --- | --- | --- | --- | --- | --- |
| *Amatitlania nigrofasciata* | competitor | - | X | - | - | X | - |
| *Amphilophus longimanus* | competitor | - | X | - | - | X | - |
| *Amphilophus rostratus* | competitor | - | - | - | - | X | - |
| *Archocentrus centrarchus* | competitor | - | - | - | - | X | - |
| *Atherinella sardina* | prey | X | X | ? | ? | X | X |
| *Dorosoma chavesii* | competitor | - | X | - | - | X | - |
| *Gobiomorus dormitor* | predator | X | X | - | ? | X | X |
| *Neetroplus nematopus* | competitor | - | X | - | - | X | - |
| *Hypsophrys nicaraguensis* | competitor | - | - | - | - | X | - |
| *Parachromis dovii* | predator | - | X | - | - | X | - |
| *Parachromis managuensis* | predator | X | X | X | X | X | X |
| *Poeciliidae* | prey | X | X | X | X | X | X |
| *Rhamdia nicaraguensis* | competitor | - | - | - | - | X | - |
| *Synbranchus marmoratus* | unclear | - | - | - | - | X | - |
| *Oreochromis spp.(tilapia)** | competitor | X | - | X | - | - | - |

* recently introduced (McCrary et al. 2007)

Given the caveats mentioned above, we did not perform formal statistical tests, but the table shows qualitatively that the extent of morphological variation in Midas cichlids is not correlated with the overall number of other fish species (e.g. L. Xiloá is inhabited by four Midas species and morphologically very diverse, but co-inhabited by 14 other fish species, whereas in the slightly more diverse L. Apoyo only five other fish species occur). We also note, that previous study (Recknagel et al. 2014) did use the number of other species (ignoring their ecological roles) as explanatory variable in regressions and found no significant correlations with the (variation in) elongation index.

Barlow, G. W. 1976. The Midas Cichlid in Nicaragua. Pp. 333-358 *in* T. B. Thorson, ed. Investigations of the ichthyology of Nicaraguan lakes. University of Nebraska Press, Lincoln.

Elmer, K. R., H. Kusche, T. K. Lehtonen, and A. Meyer. 2010. Local variation and parallel evolution: morphological and genetic diversity across a species complex of neotropical crater lake cichlid fishes. Philosophical Transactions of the Royal Society B 365:1763-1782.

Elmer, K. R., T. K. Lehtonen, S. H. Fan, and A. Meyer. 2013. Crater Lake Colonization by Neotropical Cichlid Fishes. Evolution 67:281-288.

Franchini, P., D. Monne Parera, A. F. Kautt, and A. Meyer. 2017. quaddRAD: a new high-multiplexing and PCR duplicate removal ddRAD protocol produces novel evolutionary insights in a nonradiating cichlid lineage. Mol. Ecol. 26:2783-2795.

McCrary, J. K., B. R. Murphy, J. R. Stauffer, and S. S. Hendrix. 2007. Tilapia (Teleostei: Cichlidae) status in Nicaraguan natural waters. Environ. Biol. Fishes 78:107-114.

Recknagel, H., K. R. Elmer, and A. Meyer. 2014. Crater Lake Habitat Predicts Morphological Diversity in Adaptive Radiations of Cichlid Fishes. Evolution 68:2145-2155.
